# *An Account of the Preparation, Mode of Application, and Effects, of a Liniment Recommended by* Roncalli *in the Treatment of Scrophulous Tumours*

**Published:** 1791

**Authors:** Henry Streitt

**Affiliations:** Professor of Chirurgical Pathology in the Imperial and Royal Medico-Chirurgical Academy at Vienna.


XIV. *An Account of the Preparation, Mode of Application, and Effects, of a Liniment recommended by* Roncalli *in the Treatment of ſcrophulous Tumours. By* Henry Streitt, *Profeſſor of Chirurgical Pathology in the Imperial and Royal Medico-Chirurgical Academy at Vienna.* Vide *Abhandlungen der K. K. Joſephiniſchen Medizin. Chirurg. Akademie zu Wien.* Vol. I. 4to.

THE remedy here recommended for the cure of ſcrophulous tumours was made public ſo long ago as the year 1741, by Roncalli, who obtained it from a ſurgeon at Modena; and the author of the paper before us claims only the merit of bringing it again into notice.

Roncalli deſcribes the preparation and manner of uſing it in theſe words:

"Felli bovino in ſua cyſti integro addantur "tria cochlearia ſalis communis & olei nucum: "cyſti deinde aliquo temporis intervallo, aut "ſoli, aut leviſſimo calori, expoſita. Stuppæ "ichore illo humectatæ, bis in die ſcrophuloſis "tumoribus aptentur." *

* Hiſt. Morborum. Folio, Brixiæ, 1741, p. 42.

Profeſſor

Professor Streitt observes of this liniment, that repeated trials have convinced him, first, that whenever a scrophulous tumour is capable of being dispersed, such an effect may in general be expected from the use of this application; secondly, that when the tumour cannot be dispersed, the use of the liniment serves to promote suppuration; and thirdly, that in those inveterate swellings, or species of scirrhus, which neither disperse nor come to suppuration, a long-continued use of it has been found to lessen the bulk of the tumour to a considerable degree, so as to leave only, as it were, a very small nucleus.

Ten cases are related in which this application was tried, but in the greater number of these, as the author very candidly informs us, internal remedies were occasionally administered during its use.

The subject of the first case was a soldier, nineteen years old, who had large glandular swellings in the neck and under the lower jaw.

In this case the use of the liniment was begun on the 9th of July.

On the 17th the surface of the tumour was inflamed and covered with vesicles, which made it necessary to suspend the use of the liniment till the 21ft, when it was again had recourfe to. The fwellings had now fomewhat diminifhed; but on the 9th of Auguft frefh veficles appeared, and the ufe of the liniment was obliged to be again fufpended till the 18th.

At this period of the cafe the fwellings were diminifhed to half their former bulk. The ufe of the liniment was continued without interruption from the 18th of Auguft till the 13th of September, when it was again neceffarily fufpended, during fix days, on account of the appearance of frefh veficles.

On the 30th of September the fwellings were entirely difperfed, and the patient was difcharged cured.

The internal remedies given in this cafe were, a purgative medicine, compofed of jalap and cream of tartar, at the beginning of the treatment; and afterwards a decoction of the roots of bardana and polypodium, with pills compofed of foap, gum ammoniac, and rhubarb.

The fecond patient was a foldier, aged twenty-three years, who was admitted under our author's care on the 20th of June, 1786, with a fwelling of the fubmaxillary glands of the right fide as large as a hen's egg, and feveral other other confiderable fwellings of the glands of the neck on the fame fide.

The ufe of the liniment was begun in this cafe on the 9th of July, and on the 14th the fwellings were evidently beginning to leffen and to become fofter, fo that by the end of the month they were reduced to half their former fize.

The ufe of the liniment was diligently continued during all this time, excepting, that in order to prevent veficatiqns, it was fometimes omitted, or more fparingly applied; as the ftate of the fkin, from the greater or lefs degree of rednefs, feemed to require.

Before the end of Auguft all the fwellings were difperfed except two which were ftill large though of a fofter confiftence, and thefe were entirely removed about the middle of September, on the 23d of which month the patient was difcharged cured.

The fame internal remedies were given in this as in the firft cafe.

The third patient was a foldier, aged twenty years, who had a large fwelling of the fubmaxillary gland.

He began the ufe of the liniment on the 23d of January 1787. The tumour was well rubbed rubbed with it and a pledgit previoufly moiftened with it was alfo applied to the part, and renewed twice a day; care being taken, however, to watch the ftate of the fkin, and to omit the liniment, or to leffen the quantity of it, as occafion required.

In the fpace of a month, without any affiftance from internal remedies the tumour was entirely difperfed, and on the 25th of February the patient was difcharged cured.

The fubject of the fourth cafe was a young prince, fifteen years old, who had a large fcrophulous fwelling of the glands of the neck under his right ear, of five years ftanding, which deformed his whole face. The furface of the fkin was not difcoloured, and the tumour itfelf was indolent and moveable.

In this cafe there was alfo an enlargement of the thyroid gland.

The tumours were rubbed gently every night for a quarter of an hour with the liniment, and fome of it was applied to them on pledgits and kept on all night. When it excited vefication, the ufe of it, as in the former cafes, was occafionally fufpended. In this manner the treatment was continued during three months, at the end of which time we are told the fwelling of the glands under the ear was almoſt entirely diſperſed, and that of the thyroid gland was much leſſened.

In the fifth caſe, as in that laſt related, a tumour of the parotid and ſubmaxillary glands was only partially diſperſed.

In the ſixth, which was alſo a caſe of glandular ſwelling of the neck, the tumour ſuppurated.

In the ſeventh, which was a ſwelling of the right parotid and ſubmaxillary glands, of three years ſtanding, the uſe of the liniment was perſevered in during the ſpace of five months, but at the end of that time although the ſwelling of the parotid gland is ſaid to have been almoſt entirely diſperſed, the ſubmaxillary glands were ſtill large and indurated.

In the eighth caſe the ſucceſs of the liniment was alſo incomplete; a large ſcrophulous ſwelling of the parotid and ſubmaxillary glands having been only partially leſſened by its application.

The ſubject of the ninth caſe was a ſoldier, aged twenty-five years, who had a white ſwelling of his left knee, which had been coming on ſlowly about two years, but in which there was as yet no appearance of fluctuation. Different topical applications were tried, and among others others the Hungarian remedy *, compofed of gum ammoniac and vinegar. By means of this laft the tumour feemed to be fomewhat diminifhed, but an intolerable itching made it neceffary to fufpend its ufe on the eighteenth day of the treatment. Recourfe was now had to the liniment three or four times a day, and in about fix weeks, without the affiftance of any other internal remedy than a fingle purgative medicine the fwelling was removed, and the patient, at the end of two months, was difmiffed cured.

In another patient, a man aged thirty five years, who had a fimilar complaint, which had alfo been flowly coming on, a cure was effected by the fame means in a ftill fhorter time; for at the end of five days the tumour began fenfibly to diminifh, and before another week had elapfed he was almoft entirely cured: this man took a mercurial purge twice during the treatment.

Roncalli, as we have feen, directs the liniment to be ufed only twice a day; but we find our author fometimes applying it more frequently; though he acknowledges that by fo doing the cure is not always accelerated; its

* For an account of this remedy fee the firft volume of the London Medical Journal, page 194.

operation on the ſkin when it is often repeated, rendering it neceſſary to ſuſpend its uſe from time to time, ſo that its effects in this reſpect will, of courſe, require to be particularly attended to.

